# ASCL2 is a key regulator of the proliferation–differentiation equilibrium in the esophageal epithelium

**DOI:** 10.1242/bio.059919

**Published:** 2024-01-22

**Authors:** Maude Hamilton, Zoéline Mars, Molly Sedeuil, Marjorie Rolland, Dominique Jean, François Boudreau, Véronique Giroux

**Affiliations:** ^1^ Department of Immunology and Cell Biology, Faculty of Medicine and Health Sciences, Université de Sherbrooke, Sherbrooke J1E4K8, Canada; ^2^ Centre de Recherche du Centre Hospitalier de l'Université de Sherbrooke (CRCHUS), Sherbrooke J1E4K8, Canada; ^3^ Institut de Recherche sur le Cancer de l'Université de Sherbrooke (IRCUS), Sherbrooke J1E4K8, Canada; ^4^ Université Paris Cité, Magistère Européen de génétique, Paris 75006, France

**Keywords:** ASCL2, Esophagus, Proliferation, Differentiation, Stem cells

## Abstract

The esophagus is protected from the hostile environment by a stratified epithelium, which renews rapidly. Homeostasis of this epithelium is ensured by a rare population of stem cells in the basal layer: *Keratin 15^+^* (*Krt15^+^*) cells. However, little is known about the molecular mechanisms regulating their distinct features, namely self-renewal, potency and epithelial regeneration. Achaete-scute family BHLH transcription factor 2 (ASCL2) is strongly upregulated in *Krt15^+^* stem cells and is known to contribute to stem cell maintenance in other tissues. Herein, we investigated the role of ASCL2 in maintaining homeostasis under normal and stress conditions in the esophageal epithelium. ASCL2 overexpression severely dysregulated cell differentiation and cell fate. Proliferation was also reduced due potentially to a blockage in the G1 phase of the cell cycle or an induction of quiescence. Mass spectrometry analysis confirmed alterations in several proteins associated with differentiation and the cell cycle. In addition, overexpression of ASCL2 enhanced resistance to radiation and chemotherapeutic drugs. Overall, these results denote the role of ASCL2 as a key regulator of the proliferation-differentiation equilibrium in the esophageal epithelium.

## Teaser

**Summary:** ASCL2 is a key regulator of esophageal epithelial homeostasis. ASCL2 overexpression impairs differentiation, reduces cell proliferation and enhances resistance to radiation and chemotherapeutic drugs.

## INTRODUCTION

The esophagus is protected from the austere environment by a squamous epithelium (Rosekrans et al., 2015). In mice, its flat keratinized epithelium is composed of three layers (basal, suprabasal and superficial) and renews every 7 to 10 days. Under normal conditions, cell proliferation is limited to the basal layer (Squier and Kremer, 2001). As they migrate towards the lumen, basal cells differentiate into suprabasal cells and then into superficial cells. The Notch and BMP pathways are crucial in regulating this process (Ohashi et al., 2010; Jiang et al., 2015).

A rare population of stem cells ensures the renewal of the esophageal epithelium (Giroux et al., 2017; Busslinger et al., 2021). Adult stem cells are defined by their unique capacities of self-renewal and potency (Zakrzewski et al., 2019). Some stem cell populations are also radio-resistant and/or quiescent (Hua et al., 2012; Asfaha et al., 2015; Giroux et al., 2017, 2018). In the esophagus, heterogeneity has been observed in the basal layer and the presence of stem cells has been suggested. Indeed, Cluster of differentiation 71 (CD71) and integrin α6 (Itgα6) expression was used to describe a population with a higher stemness (Croagh et al., 2007). Also, a label-retaining population expressing cluster of differentiation 34 (CD34) was shown to contribute to epithelial regeneration following injury (Kalabis et al., 2008). Finally, heterogeneity was furthermore characterized using cluster of differentiation 73 (CD73), integrin β4 (Itgβ4) and Itgα6. This study identified a Itgα6/Itgβ4^high^CD73^+^ population, which was less proliferative but had a higher clonogenic potential (DeWard et al., 2014).

The first identification of esophageal stem cells in mice was achieved using a model of lineage tracing, *Krt15-CrePR1; Rosa26^mTomato/mEGFP^* mice. *Keratin 15^+^* (*Krt15^+^*) cells are long-lived basal cells that display several characteristics of stem cells: self-renewal, regenerative potential and potency (Giroux et al., 2017). Recently, the presence of *Krt15*^+^ stem cells in the human esophageal epithelium was confirmed by single-cell RNA sequencing. Indeed, a population of quiescent stem cells was described in the basal layer: COL17A1^high^ KRT15^high^ cells (Busslinger et al., 2021). These cells would be at the head of the hierarchy as suggested by a lineage tree analysis. However, we still ignore the pathways or factors that influence the esophageal stem cell gene signature. Interestingly, an analysis of the *Krt15*^+^ mouse esophageal cells transcriptome showed that the expression of the transcription factor Achaete-scute complex homolog 2 (*Ascl2*) was highly upregulated in these cells in comparison to other basal cells (Giroux et al., 2017). Furthermore, a slight increase in *ASCL2* transcript was also reported in COL17A1^high^ KRT15^high^ cells, though statistically non-significant (Busslinger et al., 2021). Nevertheless, these results suggest that Ascl2 is overexpressed in stem/progenitor esophageal cells and could, therefore, play an important role in stemness maintenance in the esophageal epithelium.

ASCL2 is a member of the basic helix-loop-helix (bHLH) family and a gene target of the Wnt/β-catenin and Notch pathways (Moriyama et al., 2008; van der Flier et al., 2009). ASCL2 functions are tissue dependent. It has been implicated in embryonic development, differentiation, proliferation and stemness (Liu et al., 2019). During myogenesis, ASCL2 inhibits differentiation by promotor competition. In this context, ASCL2 overexpression in myoblasts also leads to the inhibition of proliferation (Wang et al., 2017). In intestinal stem cells, ASCL2 is an essential regulator of stemness in *leucine-rich repeat-containing G protein-coupled receptor 5^+^* (*Lgr5*^+^) cells. Thus, following the conditional deletion of *Ascl2*, loss of *Lgr5^+^* cells is observed in the intestine (van der Flier et al., 2009). ASCL2 is crucial for regenerating *Lgr5*^+^ cells through the dedifferentiation of progenitor cells following *Lgr5^+^* cell ablation. The deletion of *Ascl2* also prevents dedifferentiation and thereby recovery following irradiation (Murata et al., 2020).

As the role of ASCL2 remains unknown in stratified epithelia, like the esophageal epithelium, we investigated the impact of ASCL2 overexpression on self-renewal and differentiation in esophageal organoids. Our study shows that ASCL2 strongly contributes to the maintenance of the esophageal epithelium through cell differentiation and cell cycle regulation. Furthermore, it confers resistance to radiation and chemotherapeutic agents. These results suggest that ASCL2 is a key modulator of stemness in esophageal organoids.

## RESULTS

### Differentiation and morphology are altered by overexpression of ASCL2

**Fig. 1.**
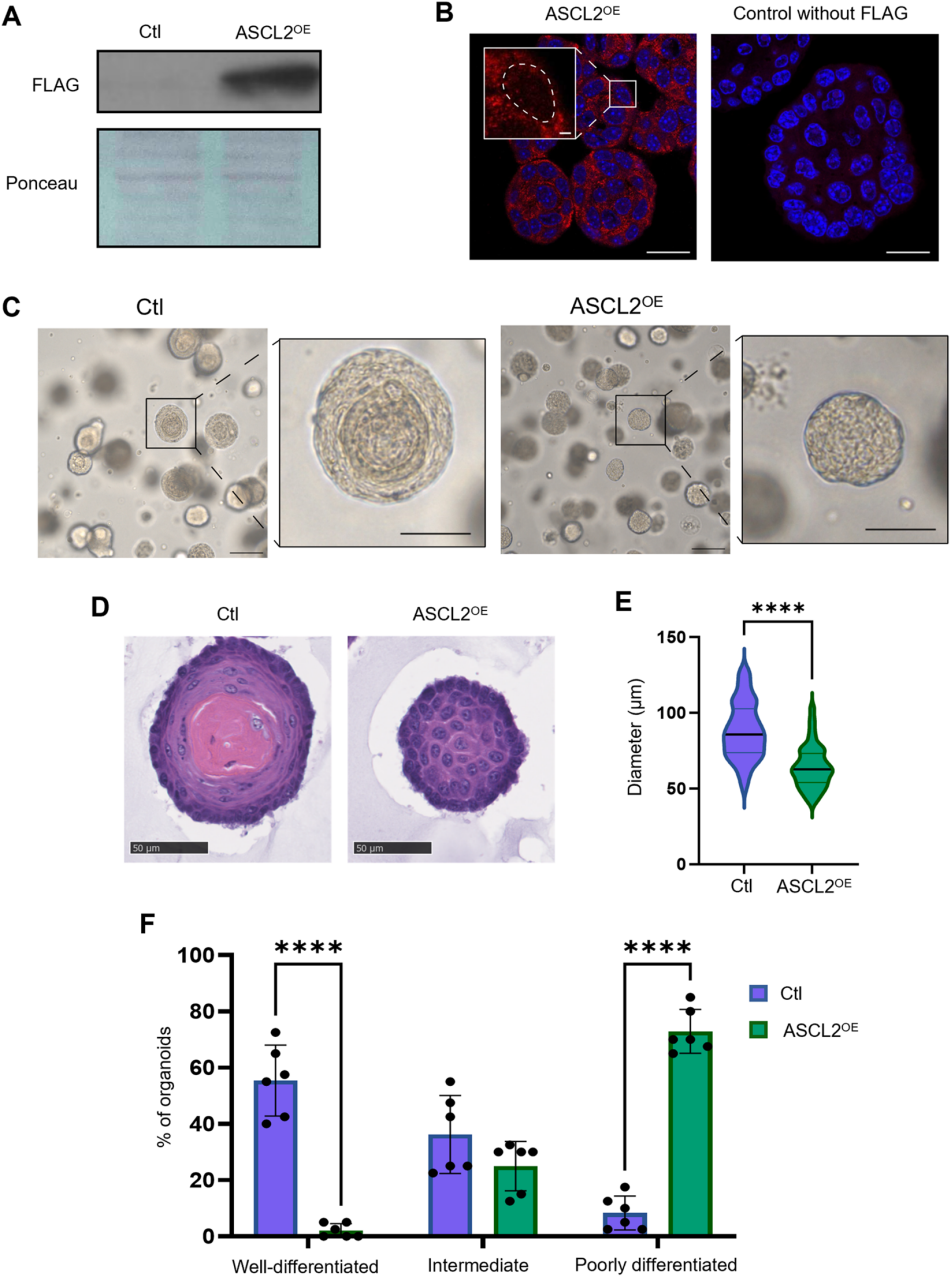
**ASCL2 overexpression alters the morphology of esophageal organoids.** (A-F) Control (Ctl) and ASCL2-overexpressing (ASCL2^OE^) esophageal organoids were obtained by lentiviral infection to express empty vector or FLAG-ASCL2, respectively. (A) Western blot confirming the overexpression of FLAG-tagged protein in the ASCL2^OE^ organoids (*n*=3). Ponceau S staining was used as the loading control. (B) Immunofluorescence was used to confirm the expression of fusion proteins (*n*=3). Scale bars: 20 µm; 2 µm (zoom-in). (C) Organoids were imaged using phase-contrast microscopy. Scale bars: 100 µm; 50 µm (zoom-in). (D) Organoids were stained with H&E to assess morphology (*n*=3). (E) Diameter was assessed on H&E coloration (*n*=3, 30 organoids per group). The violin plot shows the mean diameter and distribution values. ****P≤0.0001 using a two-tailed unpaired Welch's *t*-test. (F) Differentiation was evaluated by two investigators on H&E coloration (*n*=3, 40 organoids per group). The graph shows the percentage of organoids in each category (well differentiated, intermediate and poorly differentiated) ±s.d. ****P≤0.0001 using a two-way ANOVA test with multiple comparisons.

To investigate the role of ASCL2 in the esophageal epithelium, we generated organoids overexpressing ASCL2 (ASCL2^OE^) by lentiviral infection (Fig. 1A,B). ASCL2^OE^ organoids were significantly smaller than controls (Fig. 1E) and appeared less differentiated (Fig. 1C,D). Indeed, we observed less differentiation/keratinization by phase-contrast microscopy (dark center) and histology (pink center). Differentiation was further evaluated on Hematoxylin and Eosin (H&E)-stained sections using a scoring scale (well-differentiated, intermediate and poorly differentiated) as described in Fig. S1. Control (Ctl) organoids showed a higher percentage of well-differentiated organoids (∼53%), which were almost absent in ASCL2^OE^ organoid population (∼2%). In contrast, ASCL2^OE^ organoids were mostly poorly differentiated (∼72% versus 8% in Ctl; Fig. 1F).

**Fig. 2.**
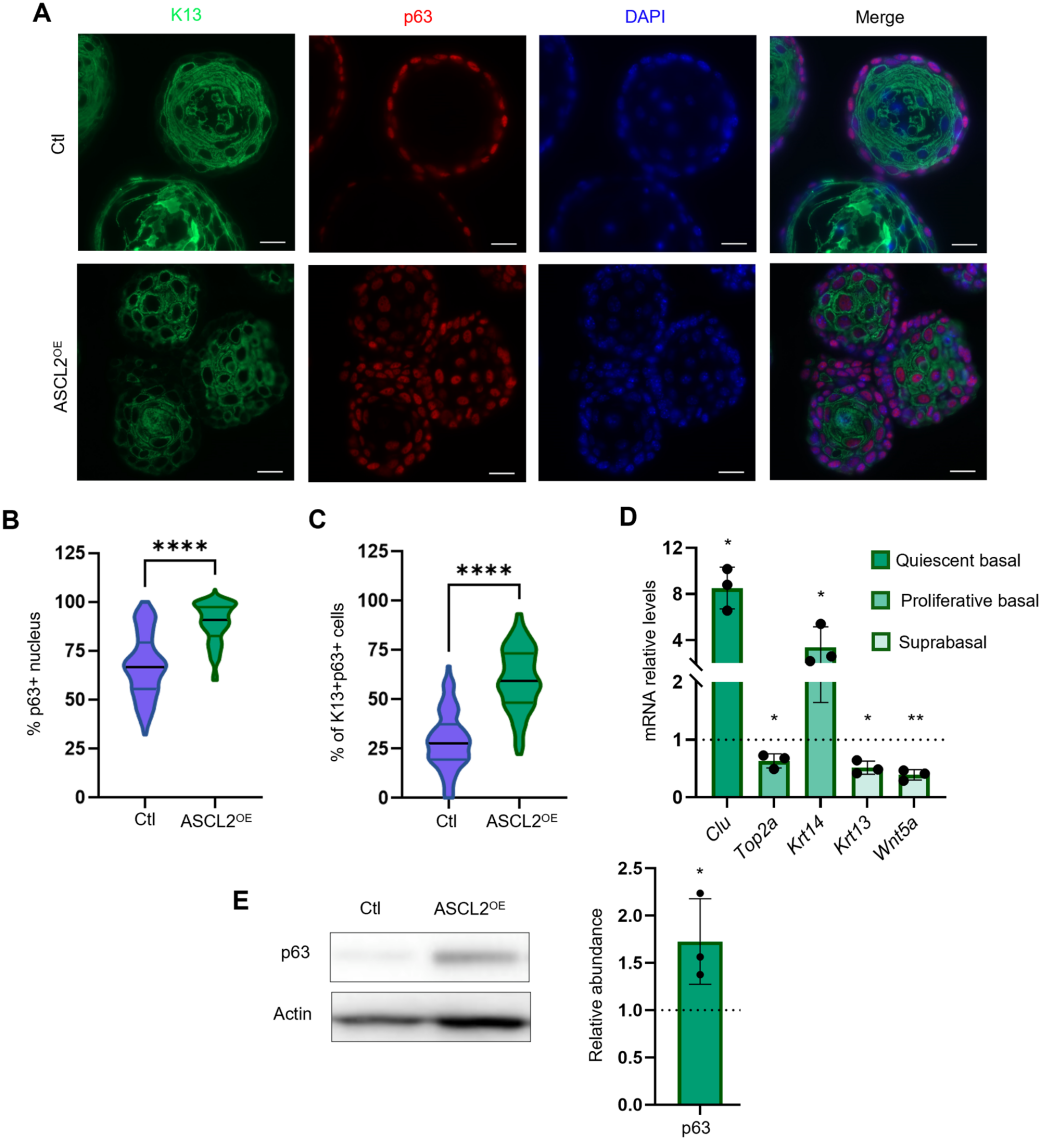
**ASCL2 overexpression in esophageal organoids causes a defect in differentiation.** (A-C) p63 (basal marker) and K13 (suprabasal marker) expression and localization were visualized by immunofluorescence (*n*=5). Scale bars: 20 µm. (B) The truncated violin plot represents the percentage of p63^+^ nuclei in organoids (*n*=5, 27-30 organoids per group). ****P≤0.0001 using a two-tailed unpaired Welch's *t*-test. (C) The truncated violin plot represents the percentage of K13^+^ p63^+^ cells by the total of p63^+^ cells (*n*=3, 27-30 organoids per group). ****P≤0.0001 using an unpaired two-tailed *t*-test. (D) Gene expression of cell subpopulation-specific markers was assessed by qPCR (*n*=3). Error bars indicate s.d. (E) p63 (basal marker) expression was assessed by western blotting. The graph represents the relative abundance (versus Ctl) ±s.d. (*n*=3). *P≤0.05, **P≤0.01 using a two-tailed unpaired Welch's *t*-test.

Differentiation was furthermore explored using p63 as a marker for basal cells and K13 for suprabasal (differentiated) cells. In ASCL2^OE^ organoids, immunofluorescence revealed that the number of p63^+^ cells was increased (Fig. 2A,B), which was confirmed by western blotting (Fig. 2E). Surprisingly, p63^+^ cells were also present outside of the basal layer, i.e. the periphery of the organoid (Fig. 2A, second panel). Interestingly, double-positive cells (K13^+^ p63^+^) were more prominent in ASCL2^OE^ organoids (Fig. 2C). Thus, cells seem to remain in an intermediate state of differentiation, as they expressed both basal (p63^+^) and suprabasal (K13^+^) markers. mRNA levels of known suprabasal markers, *Krt13* and *Wnt5a* (Busslinger et al., 2021), were reduced in ASCL2^OE^ organoids, while levels of the basal markers *Clu* and *Krt14* were increased (Fig. 2D). All these results demonstrate that the morphological changes in ASCL2^OE^ organoids result, in part, from impaired differentiation.

### Proliferation and clonogenic potential are reduced in ASCL2^OE^ organoids

**Fig. 3.**
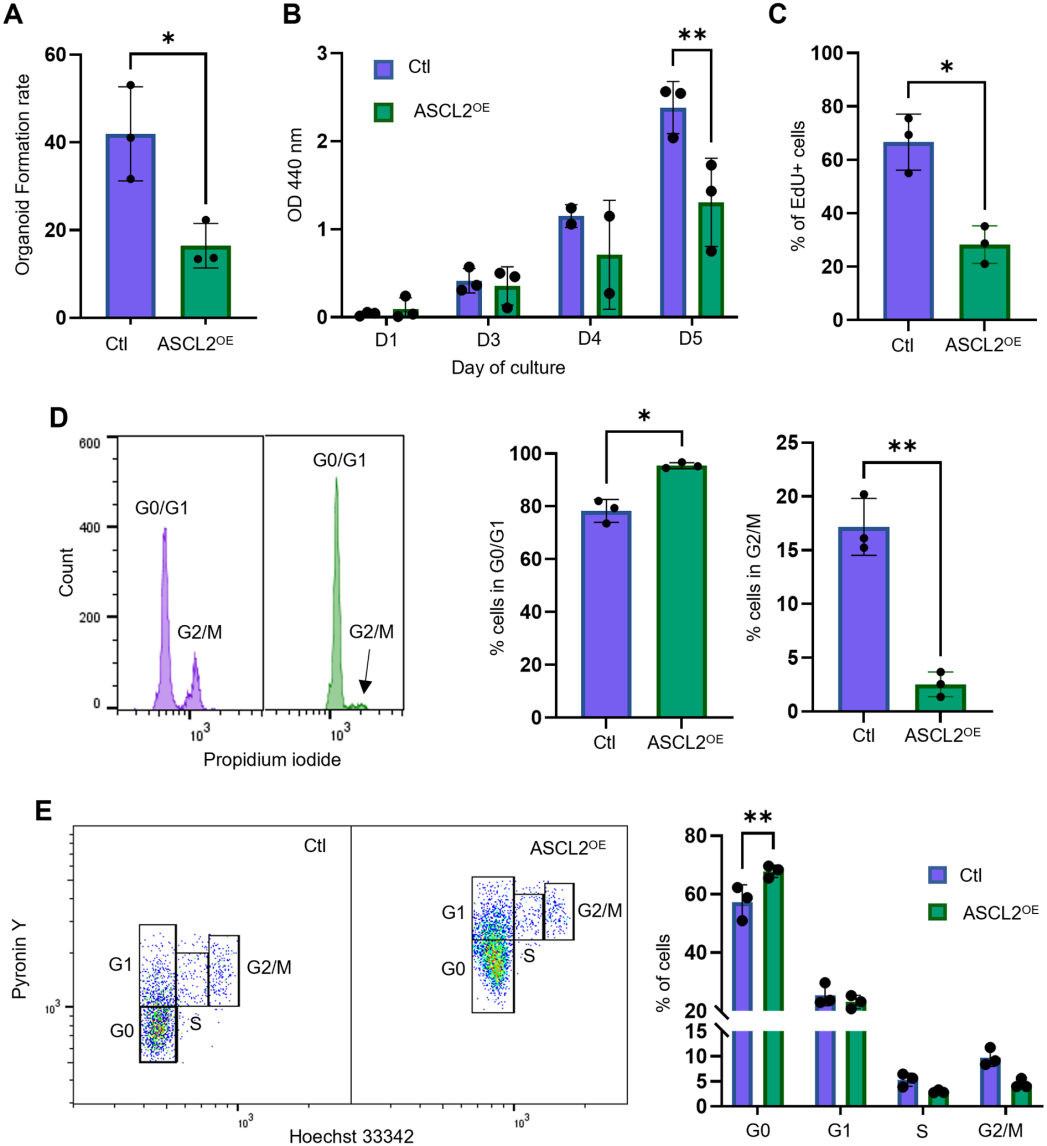
**Proliferation, clonogenicity and cell cycle progression of esophageal epithelial cells are dysregulated in organoids overexpressing ASCL2.** (A) Organoid formation rate was assessed in each group (*n*=3). The graph represents the number of organoids formed from 300 seeded cells ±s.d. *P≤0.05 using an unpaired two-tailed *t*-test. (B) Proliferation was assessed at days 1, 3, 4 and 5 post seeding using the WST-1 assay (*n*=3). The graph represents mean±s.d. **P≤0.01 using a two-way ANOVA with multiple comparisons. (C) Proliferation was assessed by quantification of proliferative (EdU^+^) cells by flow cytometry. The graph represents mean±s.d. (*n*=3). *P≤0.05 using a paired two-tailed *t*-test. (D) Cell cycle analysis of organoids was performed using propidium iodide. The graphs represent the mean of the percentage of cells in G0/G1 or in G2/M ±s.d. (*n*=3). *P≤0.05, **P≤0.01 using a paired two-tailed *t*-test. (E) Cell cycle analysis was performed using Pyronin Y and Hoechst 33342 staining. Representative results are shown. The graph represents the mean of the percentage of cells in each phase ±s.d. (*n*=3 independent experiments). **P≤0.01 using a two-way ANOVA with multiple comparisons.

Epithelial homeostasis requires fine-tuning between differentiation and proliferation. As ASCL2 could be a key regulator of cell differentiation, we also investigated the impact of its overexpression on proliferation and clonogenicity. First, to compare the clonogenic potential of ASCL2^OE^ cells, we quantified the organoid formation rate, i.e. the proportion of cells able to form organoids. ASCL2^OE^ cells had a lower capacity to form organoids than Ctl cells (Fig. 3A). Moreover, WST-1 assays suggested a reduction of proliferation in ASCL2^OE^ cells (Fig. 3B). This result was confirmed by 5-ethynyl-2′-deoxyuridine (EdU) incorporation assay. There were ∼38% less EdU^+^ cells in ASCL2^OE^ organoids compared to those in Ctl organoids (Fig. 3C). In agreement with these results, mRNA levels of *Top2a*, a known proliferation marker (Busslinger et al., 2021), were significantly decreased in ASCL2^OE^ organoids (Fig. 2D). We validated that these observations were not attributed to changes in cell viability (Fig. S2). These results altogether suggest that ASCL2 could be involved in regulating the clonogenic and proliferative potential of esophageal epithelial cells.

### Cell cycle progression is dysregulated with overexpression of ASCL2

Given that proliferation and clonogenicity of ASCL2^OE^ cells were reduced in organoids, we hypothesized that progression through the cell cycle might be impaired. A more quiescent phenotype could also explain a decrease in proliferation. Dissociated cells of organoids were stained with propidium iodide to assess cell cycle phases. Interestingly, there was an important decrease in the percentage of cells in G2/M (17.18% for Ctl cells versus 2.53% for ASCL2^OE^ cells, Fig. 3D). In return, there were more cells in G0/G1 in the ASCL2^OE^ cell population. As the baseline of Hoechst and propidium iodide was not the same in both of our organoid lines, we treated organoids 24 h prior harvesting with 100 ng/ml of nocodazole, an inhibitor of the cell cycle that causes arrest in G2/M, to confirm the gating strategy for each cell cycle phase (Fig. S3). To further explore cell cycle progression in our model, we stained organoid cells with the DNA stain Hoechst 33342 and the RNA stain Pyronin Y, which allow the discrimination of cells in G0, G1, S and G2/M. Our results revealed that there was an ∼10% increase in the number of cells in G0 for the ASCL2^OE^ population, suggesting a possible effect of ASCL2 on quiescence (Fig. 3E). Interestingly, mRNA expression of a known marker of quiescent cells, *Clu*, was considerably increased in ASCL2^OE^ organoids (Fig. 2D). Thus, reduced proliferation and clonogenicity in ASCL2^OE^ organoids could be the result of changes in cell cycle progression with an increase in the number of quiescent cells.

### Proteomic analysis of ASCL2^OE^ organoids

**Fig. 4.**
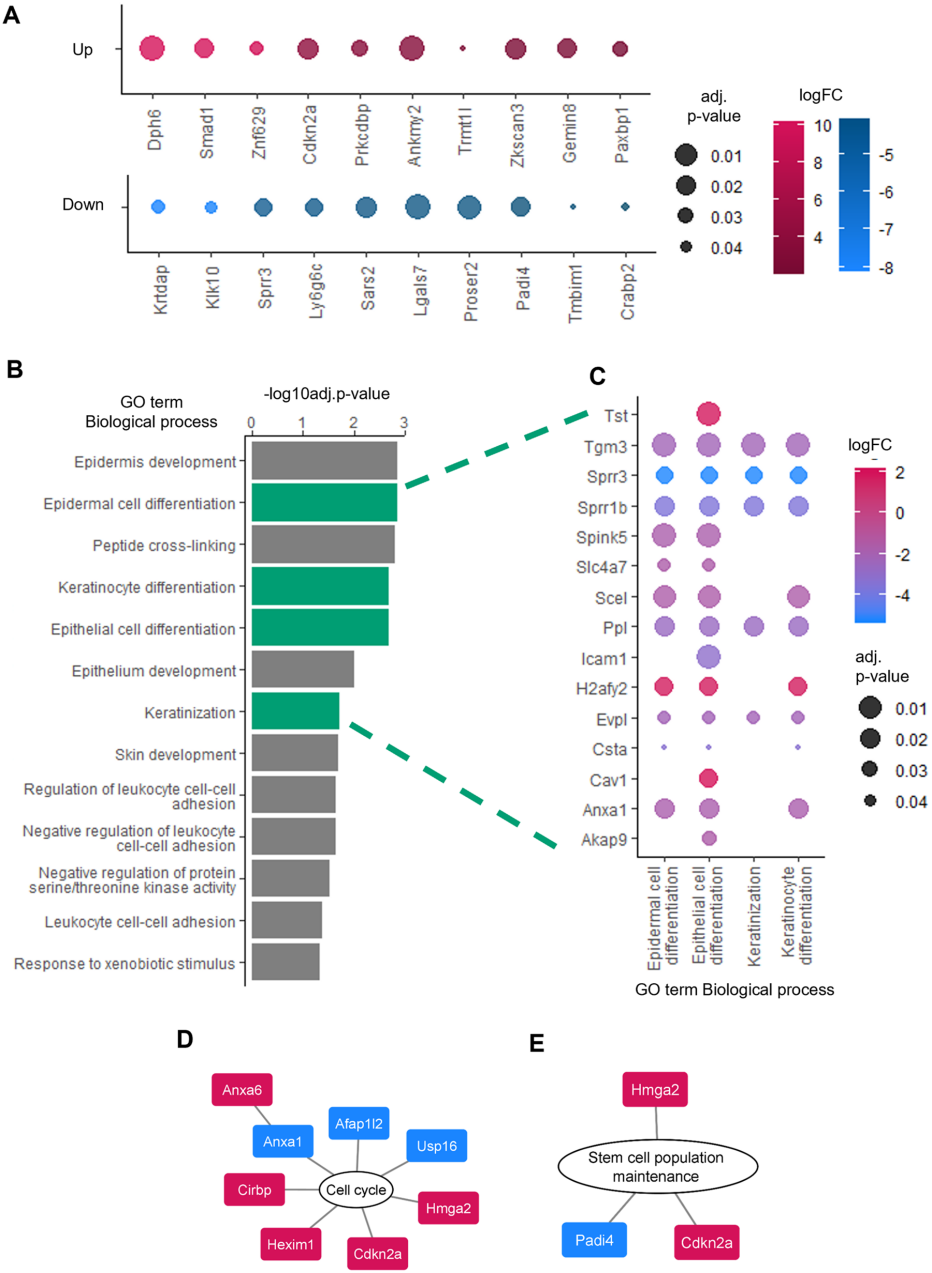
**Mass spectrometry analysis comparing Ctl and ASCL2^OE^ organoids confirms differentiation and cell cycle impairment.** (A-F) Mass spectrometry was performed on total protein extracts of Ctl and ASCL2^OE^ organoids (*n*=3). The results were analyzed using iPathwayGuide (Advaita Bio). Upregulated proteins are shown in magenta and downregulated proteins in blue. (A) The ten most upregulated and downregulated proteins are shown. The color of the dots represents log fold change (logFC) and the dot size represents adjusted *P*-value (adj. *P*-value). (B) GO terms related to biological processes over-represented in the ASCL2^OE^/Ctl comparison are illustrated. Graph represents –log (base 10) of adj. *P*-value with FDR correction, bars in green represent GO terms related to differentiation. (C) Proteins differentially expressed with GO terms related to differentiation processes are shown. Dot color represents logFC and dot size represents adj. *P*-value. (D,E) Proteins significantly regulated that are related to the cell cycle and stem cell population maintenance are illustrated.

To determine the molecular changes occurring following ASCL2 overexpression, we performed data-independent acquisition (DIA) mass spectrometry (MS) on both organoid populations. We identified 37 upregulated and 57 downregulated proteins in ASCL2^OE^ organoids compared to Ctl organoids. Of note, keratinocyte differentiation-associated protein (KRTDAP), a protein responsible for the regulation of keratinocyte differentiation and maintenance of stratified epithelia (Oomizu et al., 2000), was the most downregulated protein in ASCL2^OE^ organoids (Fig. 4A). Gene Ontology (GO) analysis revealed that several biological processes related to differentiation (epidermal cell, keratinocyte and epithelial cell differentiation) and keratinization were over-represented in our dataset (Fig. 4B). Several proteins associated with differentiation were differentially expressed in ASCL2^OE^ organoids, such as small proline-rich protein 3 (SPRR3) and macroH2A.2 histone (H2AFY2; Fig. 4C). As ASCL2 is a transcription factor, we performed alignment of its motif on the promoter of the genes encoding for the ten most downregulated or upregulated proteins in our proteomic results. Three of these genes, *Ly6g5c*, *Tmbim1* and *Crabp2*, showed putative binding sites for ASCL2 in their 500 bp promoter region (Fig. S5). On the other hand, proteins related to the cell cycle were also modulated in ASCL2^OE^ organoids (Fig. 4D). p16^INK4a^ (Cdkn2a), a protein implicated in cell cycle progression and capable of inducing cell cycle arrest (Medema et al., 1995), was the fourth most upregulated protein in our dataset (Fig. 4A). p16^INK4a^ and SMAD1 upregulation were confirmed by western blotting (Fig. S4). Interestingly, SMAD1 is involved in the BMP pathway, which is crucial for esophageal development and homeostasis. Some proteins associated with stem cell population maintenance were also significantly regulated in ASCL2^OE^ organoids, suggesting that ASCL2 could be involved in stemness as well (Fig. 4E). These molecular changes in ASCL2^OE^ organoids strongly correlate with the biological changes observed, confirming that ASCL2 plays an important role in differentiation and the cell cycle in esophageal organoids.

### ASCL2 overexpression confers radio- and chemo-resistance

**Fig. 5.**
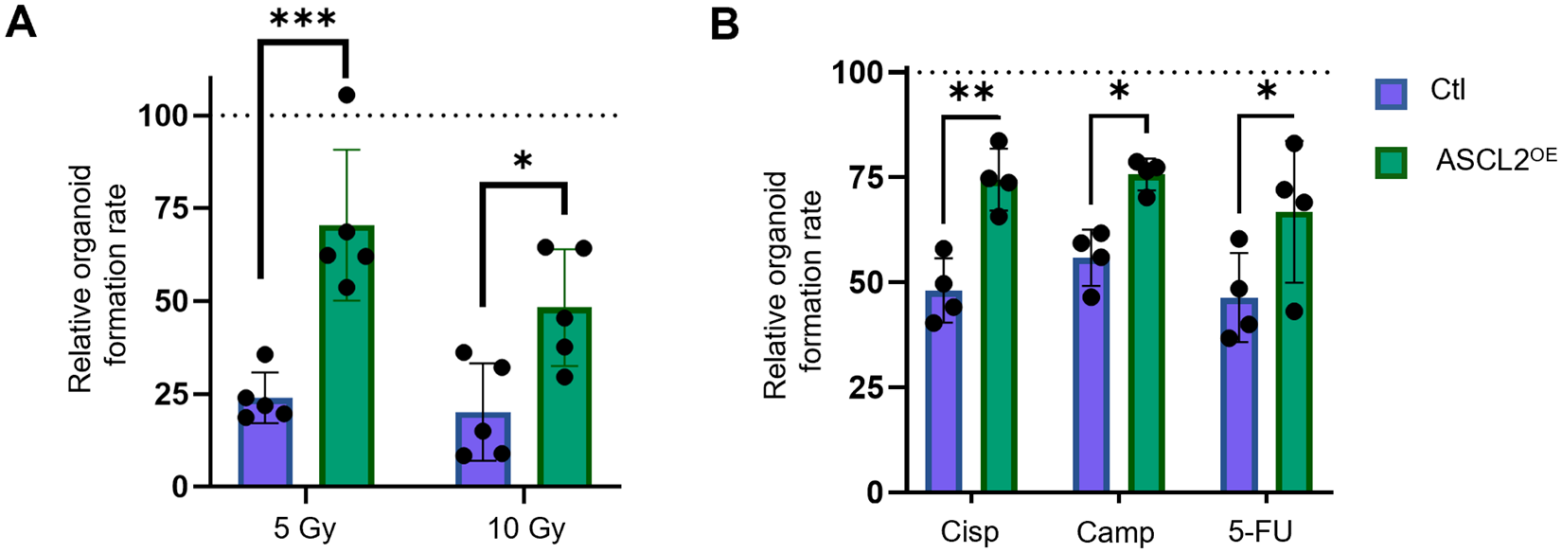
**ASCL2^OE^ organoids display radio- and chemo-resistance capacities.** (A,B) Organoid formation rate was assessed in each group following irradiation (5 or 10 Gy; *n*=5) or treatment with chemotherapy drugs [cisplatin (Cisp), camptothecin (Camp) or 5-FU; *n*=4]. Graphs represent the relative organoid formation rate (versus non-irradiated or untreated controls) ±s.d. *P≤0.05, **P≤0.01 and ***P≤0.001 using a two-way ANOVA with multiple comparisons.

ASCL2 regulates several functions associated with stem cells, namely, cell proliferation and differentiation. Therefore, we investigated the contribution of ASCL2 to another key characteristic of stem cells, resistance to anti-cancer treatments. To do so, organoids were irradiated with 5 or 10 Gy on day 1 post-seeding, and the number of organoids formed was evaluated. As expected, both populations showed reduced organoid formation rate compared to that of their non-irradiated control (Fig. 5A). Particularly, ASCL2^OE^ organoids maintained a higher capacity to form organoids following 5 or 10 Gy irradiation than that of controls. We also tested chemo-resistance in our organoid model. Organoids were treated with cisplatin, camptothecin or 5-fluorouracil (5-FU) at day 1 and the number of organoids was counted at day 7. Interestingly, more organoids were formed in the ASCL2^OE^ organoid line following each treatment than in controls (Fig. 5B). In conclusion, these results showed that ASCL2 overexpression confers radio- and chemo-resistance capacities to esophageal epithelial cells.

## DISCUSSION

Epithelial homeostasis is typically assured by stem cells. Renowned for their high self-renewal capacity and potency, this subpopulation of epithelial cells also contributes to tissue regeneration in response to injury. In the esophagus, the presence of a true stem cell population remained controversial until recently (Croagh et al., 2007, 2008; Doupé et al., 2012; DeWard et al., 2014). In 2017, *Krt15^+^* esophageal stem cells were identified in mice (Giroux et al., 2017), and their presence was recently confirmed in humans as quiescent COL17A1^high^ KRT15^high^ cells (Busslinger et al., 2021). However, we still ignore the molecular players dictating their distinct features. Herein, we investigated the role of ASCL2, which was previously identified as the most upregulated transcription factor in *Krt15^+^* stem cells (Giroux et al., 2017), using esophageal organoids. Organoid culture is the predilected model to study functions associated with stemness, such as clonogenicity, differentiation and cell proliferation, as it maintains stem cell growth.

Upon ASCL2 overexpression, differentiation was severely impaired in organoids. Keratinization, a sign of terminal differentiation in the mouse esophagus, was drastically reduced histologically. Proteomic data also supported dysregulation in biological processes related to keratinization and differentiation. ASCL2 can act as a transcriptional activator but also as an inhibitor. Sequence alignment suggests putative binding sites for ASCL2 on the promoter regions of the genes encoding for three downregulated proteins. One of those proteins, Crabp2, is implicated in the transport of retinoic acid to the nucleus and promotes differentiation (DeWard et al., 2014). These observations corroborate other studies reporting a role for ASCL2 in the differentiation of trophoblast cells, myoblasts and lymphocytes (Liu et al., 2014; Wang et al., 2017; Bogutz et al., 2018; Bao et al., 2020; Varberg et al., 2021). Interestingly, ASCL2 was also recently associated with the dedifferentiation of intestinal crypt cells in response to stem cell ablation to cope with the injury and induce regeneration (Murata et al., 2020). The differentiation changes observed in our study were confirmed by staining for specific markers of basal and suprabasal cells. An enhanced number of basal (p63^+^) cells was observed in ASCL2^OE^ organoids as well as an aberrant presence of cells expressing basal (p63) and suprabasal (K13) cell markers. This observation suggests an impairment in cell fate decisions in these organoids. Of note, ASCL2 was shown to regulate cell fate in the intestine (van der Flier et al., 2009; Giakountis et al., 2016) and in embryonic stem cells (Kinoshita et al., 2021). In the normal esophageal epithelium, cells migrate toward the lumen while undergoing differentiation from basal to suprabasal cells (Rosekrans et al., 2015). Herein, a large proportion of cells remains in an intermediate state, suggesting that terminal differentiation is dysregulated and that cell fate is compromised.

As alterations in cell fate decisions might impact clonogenic potential, we evaluated the organoid formation rate and it was indeed reduced in ASCL2^OE^ organoids. In accordance with decreased clonogenicity, ASCL2 overexpression reduced cell proliferation in esophageal organoids. ASCL2 has been previously associated with reduced cell proliferation in Schwann cells, myoblasts and neural stem cells (Küry et al., 2002; Wang et al., 2017; Liu et al., 2019). However, in the small intestine, ASCL2 supports a high proliferation (van der Flier et al., 2009; Schuijers et al., 2015). This difference could be due to the higher dependency of intestinal tissue to the Wnt/β-catenin pathway than that of esophageal tissue, amongst others. The reduced proliferation observed in ASCL2^OE^ organoids correlated with a lower number of cells in G2/M and an increase of cells in G1/G0, suggesting a blockage in cell cycle progression. Notably, proteomic data showed an increased expression of p16^INK4A^ (Cdkn2a) in ASCL2^OE^ organoids compared to that in controls. This cell cycle inhibitor leads to an arrest in the G1 phase of the cell cycle (Medema et al., 1995), which could explain our observations. Furthermore, Pyronin Y/Hoechst 33342 staining showed an increase in G0 cells, suggesting induction of quiescence following ASCL2 overexpression. Quantitative PCR (qPCR) results also showed induced expression of *Clu*, a gene enriched in quiescent basal cells in the human esophagus (Busslinger et al., 2021). High expression of *Clu* was also observed in a damage-induced quiescent cell population in the gut (Ayyaz et al., 2019). Overall, in response to ASCL2 overexpression, organoids are less clonogenic, proliferative, differentiated and potentially more quiescent, suggesting again a major impairment in cell fate.

Cell fate decision is mainly associated with stem cells. Of note, proteins associated with stem cell maintenance were regulated in ASCL2^OE^ organoids. Another distinct feature of stem cells is a higher capacity to cope with stressors such as radiation and chemotherapeutic drugs. In the esophagus, *Krt15^+^* stem cells were shown to be resistant to high-dose radiation (Giroux et al., 2017), so we would expect higher resistance in ASCL2^OE^ organoids as well. Indeed, ASCL2 overexpression reduced sensitivity to radiation. Higher resistance to three chemotherapeutic agents (5-FU, cisplatin and camptothecin) was also observed with ASCL2^OE^ organoids. Aberrant upregulation of ASCL2 was previously associated with 5-FU resistance in gastric cancer (Kwon et al., 2013). In addition, ASCL2 was linked to drug resistance in colorectal cancer cells (Tanaka et al., 2019). More recently, high expression of ASCL2 in esophageal adenocarcinoma was proposed as a poor prognosis of survival (Shibahara et al., 2022). Interestingly, proteomic analysis of ASCL2^OE^ organoids showed altered expression of SPRR3, a protein that has been associated with resistance to DNA damage inducers in esophageal cancer cells (Luo et al., 2013).

Overall, our results demonstrate that ASCL2 contributes to epithelial homeostasis as a critical regulator of the differentiation-proliferation balance. Indeed, ASCL2^OE^ organoids displayed dysregulation in differentiation, cell proliferation, clonogenicity and the cell cycle. These various alterations suggest impaired cell fate decisions. These phenotypes correlated with proteomic changes in biological processes associated with these functions. Furthermore, ASCL2^OE^ organoids were also more resistant to radiation and chemotherapeutic drugs, suggesting a role for ASCL2 in maintaining homeostasis and in response to injury. Further studies will be required to define how ASCL2 mechanistically regulates these pathways. Inactive DNA-binding ASCL2 and ASCL2 derivatives with specific mutations could be used to mechanistically underline the phenotypes observed in ASCL2^OE^ organoids. This study highlights a new role for ASCL2 in the esophageal epithelium, contributing to our understanding of the molecular mechanisms involved in the fine-tuning of the homeostasis in this epithelium.

## MATERIALS AND METHODS

### Organoid generation and culture

Organoids were established from the esophageal epithelium of wild-type 8-week-old C57BL/6J mice as described previously (Giroux et al., 2017). All animal procedures were approved by the Université de Sherbrooke Institutional Committee for the Use and Care of Animals. Briefly, the esophagus was open longitudinally and incubated in dispase (CB-40235, ThermoFisher Scientific). The epithelium was then peeled from the submucosa and incubated in trypsin to obtain a single-cell suspension. Isolated cells were then seeded in Matrigel™ (354230, Corning) and grown as organoids in organoid medium [Advanced DMEM/F12 (12634010, Gibco) supplemented with 1× GlutaMAX™ (35050061, ThermoFisher Scientific), 1× HEPES (330-050-EL, Wisent), 1× penicillin-streptomycin (450-201-EL, Wisent), 1× N2 supplement (17502048, ThermoFisher Scientific), 1× B27 supplement (17504044, ThermoFisher Scientific), 0.1 mM N-acetylcysteine (A7250-5G, Sigma-Aldrich), 50 ng/ml recombinant EGF (315-09, PeproTech), Noggin/R-Spondin homemade conditioned medium and 10 µM Y27632 (A11001-50, AdooQ Bioscience)]. The medium was changed every other day, and organoids were passaged after 7 days of culture. For harvesting or passaging, organoids were retrieved from Matrigel™ using dispase. At this step, organoids were fixed in zinc formalin for 4 h at 4°C for histology or used for protein or RNA extraction. For passaging, organoids were further dissociated using trypsin and filtered using a 40 µm cell strainer to obtain a single-cell suspension. Cells were counted and seeded again in Matrigel™. Organoids were regularly tested for mycoplasma contamination.

### Lentiviral construction and infection in organoids

The Gateway method was used to generate lentiviral construction for protein overexpression (FLAG-ASCL2). The sequence was synthesized and cloned in pUC57 vectors (Bio Basic; Markham, Ontario, Canada). pDONR221 was used as a donor vector (graciously provided by Professor François-Michel Boisvert, Université de Sherbrooke) and pLX303 as a destination vector (graciously provided by Professor Marie-Josée Boucher, Université de Sherbrooke). The pUC57 vector containing the gene of interest was digested using the restriction enzymes BamH1 (R3136S, New England Biolabs) and Nde1 (R0111S, New England Biolabs). The purified insert [purification kit Monarch (T1020S, New England Biolabs), following the supplier's recommendations] was recombined in donor vectors by BP recombination. Afterward, the vector was transformed in Stellar™ competent bacteria (636766, Takara Bio). Bacteria were seeded on agar Petri dishes with kanamycin and, following amplification of positive colonies, the vector was extracted by miniprep using the GeneJetPlasmid Miniprep kit (K0503, ThermoFisher Scientific) following the supplier's recommendations. LR recombination was completed using pDONR221 vectors containing the gene of interest and the pLX303 destination vector. Those are already listed as gifted in the beginning of the paragraph. The vector was transformed in One Shot™ Stbl3™ competent bacteria (C737303, Invitrogen), positive colonies were amplified, and the vector was extracted using the QIAfilter Plasmid Midi kit (12243, Qiagen). Plasmids were validated by sequencing.

Overexpressing (ASCL2^OE^) and Ctl (pLX303-empty) organoid lines were obtained by lentiviral infection. Viruses were produced in HEK293T cells by transfection (Lipofectamine™ 3000; L3000015, ThermoFisher Scientific), harvested after 48 h in the supernatant and filtered using a 0.45 µm filter. The viral solution was concentrated overnight using Lenti-X™ concentrator (one volume of Lenti-X™ for three volumes of supernatant; 631231, Takara Bio) according to the manufacturer's recommendations. 20,000 organoid cells (single-cell suspension) were incubated for 1 h at room temperature for Spinfection with concentrated virus and polybrene (H9268, Sigma-Aldrich). The supernatant was then removed and cells were seeded in Matrigel™ for 7 days of culture. At day 7, organoids were passaged and antibiotic selection with blasticidin (R21001, ThermoFisher Scientific) was performed for 7 days.

### Western blotting

Organoids were lysed using RIPA buffer supplemented with protease and phosphatase inhibitors. Samples were flown through a 27G needle several times, incubated for 10 min on ice and then sonicated at 5 s intervals at 20% amplitude for 30 s. Following centrifugation, the supernatant was recovered. Protein extracts were quantified using the bicinchoninic acid (BCA) method, diluted in Laemmli buffer and then incubated for 5 min at 95°C. Proteins were separated on SDS-PAGE gels and then transferred to polyvinylidene fluoride (PVDF) membranes. Transfer and protein loading were confirmed by Ponceau Red staining. The membrane was blocked with milk [5% powdered milk in PBS/Tween 0.05% (PBS/T)] or 5% BSA in PBS/T for 1 h at room temperature, and then incubated overnight at 4°C in primary antibody diluted in blocking solution (see Table 1). The membrane was then incubated 1 h at room temperature with HRP-coupled secondary antibodies. The signal was visualized using the Enhanced Chemiluminescence kit (ECL; RPN2232, VWR or 1705062S Bio-Rad) and observed by autoradiography or a chemiluminescent imaging system (Azure 280, VWR). Uncropped images of western blots are shown in Fig. S6.

**Table 1.**
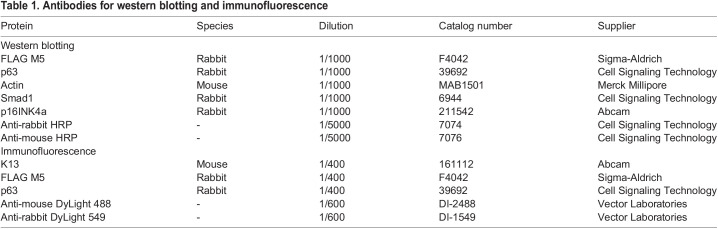
Antibodies for western blotting and immunofluorescence

### Histological analysis and immunofluorescence

Following zinc formalin fixation, organoids were resuspended in a plug of 2.5% gelatine/2% agar. Following circulation and paraffin embedding, specimens were sectioned (4-5 µm) using a Histocore Multicut microtome (Leica). For histological analysis, H&E coloration was then performed. Slides were imaged using a slide scanner (Hamamatsu Nanozoomer 2.0-RS). The diameter of organoids was measured using NDP.view2 software (Hamamatsu). Differentiation was evaluated using the following criteria by two unbiased investigators: well-differentiated (normal differentiation with a keratinized center), intermediate (basal and suprabasal layers) and poorly differentiated (overall basal cell organoids) (Fig. S1). For immunofluorescence, slides were rehydrated and antigens were retrieved in citric acid. Slides were blocked using 5% BSA and incubated overnight at 4°C in primary antibodies diluted in 1% BSA solution (see Table 1). They were then incubated for 1 h at room temperature with secondary antibodies diluted in 1% BSA solution. Sections were mounted using VECTASHIELD^®^ (H-1200, Vector Laboratories) with DAPI. Slides were then imaged on a Zeiss Axioscope upright microscope or a confocal Zeiss LSM 880 two-photon microscope.

### RNA extraction and qPCR

RNA was extracted using the RNeasy Plus Mini kit (74134, Qiagen) following the supplier's recommendations. 1 µg of RNA was then reverse transcribed using SuperScript III Reverse Transcriptase (18080-044, ThermoFisher Scientific) and oligo-dT_12-18_ according to the manufacturer's recommendations. FastStart Essential DNA Green Master (06402712001, Roche) was used for qPCR analysis on a LightCycler^®^ 480 (Roche). Primers used can be found in Table 2. Results were calculated in normalized ratio using the reference gene *Gapdh.*

**Table 2.**
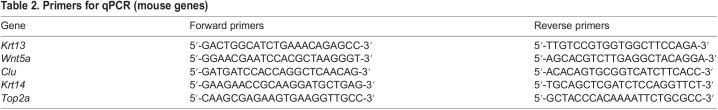
Primers for qPCR (mouse genes)

### Organoid formation rate

When organoids were passaged, 300 cells were seeded in 20 µl of Matrigel™ in a 48-well plate. Organoids were cultured as described above. Assays were done in quadruplicate for each condition and the number of organoids formed was counted 7 days post seeding. For the radio-resistance assay, organoids were irradiated at day 1 post seeding with 5 or 10 Gy and non-irradiated organoids were used as controls. For the chemo-resistance assay, organoids were incubated at day 1 post seeding with chemotherapeutic agents (30 μM cisplatin, 13119, Cayman Chemicals; 50 nM camptothecin, 11694, Cayman Chemicals; 10 μM 5-FU, 14416, Cayman Chemicals) or vehicle for 36 h.

### Proliferation assays

For the WST-1 assay, when organoids were passaged, 1000 cells were seeded in 5 µl of Matrigel™ in a 96-well plate. Organoids were cultured as described above. Assays were done in quadruplicate for each condition and a negative control containing no cells was prepared. Proliferation was evaluated using the WST-1 reagent (5015944001, Sigma-Aldrich) according to the manufacturer's recommendations. The absorbance was measured using a plate reader. For EdU incorporation assay, 20,000 cells were seeded per well in a 48-well plate. At day 4 post seeding, organoids were incubated with 10 μM of EdU (C10419, ThermoFisher Scientific) for 16 h. Organoids were then harvested and dissociated to obtain a single-cell suspension. Cells were stained for EdU using the Click-iT™ EdU Alexa Fluor™ 647 Flow Cytometry Assay Kit (C10419, ThermoFisher Scientific) according to the manufacturer's instructions. EdU^+^ cells were quantified using a BD Fortessa flow cytometer and FlowJo software.

### Cell cycle assay

The cell cycle was assessed by RNA and DNA staining. Organoids were harvested at 7 days post seeding and dissociated to a single-cell suspension. Cells were fixed for least 2 h using 70% ethanol. Cells were then stained (50 μg/ml propidium iodide, P1304MP, ThermoFisher Scientific; 4 μg/ml Pyronin Y, 418630010, ThermoFisher Scientific; 2 μg/ml Hoechst 33342, H3570, ThermoFisher Scientific) and the cell cycle phase was assessed using a BD Fortessa flow cytometer and FlowJo software.

### Digestion of total protein extract for DIA-MS

Ctl and ASCL2^OE^ organoids were extracted in a solution of 8 M urea, 1 M NH_4_HCO_3_ and 20 mM HEPES, pH 8.0. Then, the samples were sonicated on ice at 5 s intervals at 20% amplitude for 60 s and centrifuged at 16,000 ***g*** for 10 min at 4°C. 30 μg of proteins was reduced by adding dithiothreitol (final concentration of 5 mM). Samples were incubated for 2 min at 95°C, followed by incubation at 30 min at room temperature. Protein alkylation was completed by the addition of chloroacetamide (final concentration of 7.5 mM; Sigma-Aldrich) for 20 min in the dark at room temperature. Urea concentration was diluted (final concentration of 2 M) by adding 50 mM NH_4_HCO_3_ (Sigma-Aldrich). 1 μg of MS-grade trypsin (ThermoFisher Scientific) was added to each sample for protein digestion and incubated overnight at 30°C with agitation.

### Purification and desalting of peptides on C18 Columns for DIA-MS

Digestion was stopped with trifluoroacetic acid (final concentration of 0.2%; Sigma-Aldrich). Digested peptides were purified with C18 tips (ThermoFisher Scientific Pierce) according to the manufacturer's recommendations. Using a centrifugal evaporator, samples were dried at 60°C for at least 3 h and resuspended in 1% formic acid (FA).

### Fractionation of digested samples for the DIA library

Samples were combined to create the DIA library, which was fractionated using the Pierce High pH Reversed-Phase Peptide Fractionation Kit (84868, ThermoFisher Scientific) according to the manufacturer's recommendations. Peptides were eluted in eight fractions and dried using a centrifugal evaporator at 60°C for at least 3 h. Samples were then resuspended in 1% FA.

### Data-dependent acquisition (DDA) liquid chromatography (LC)-MS analysis for DIA library

All mass spectrometry experiments were performed at the Mass spectrometry and Proteomics platform at Université de Sherbrooke. From each fraction, 250 ng of peptides were injected into an HPLC (nanoElute, Bruker Daltonics) and loaded onto a trap column (constant flow of 4 µl/min; Acclaim PepMap100 C18 column, 0.3 mm internal diameter×5 mm, Dionex Corporation). An analytical C18 column (1.9 µm beads size, 75 µm×25 cm, PepSep) was used for elution for over a 2 h gradient of acetonitrile (5-37%) in 0.1% FA at 400 nl/min while being injected into a TimsTOF Pro trapped ion mobility mass spectrometer (TIMS) equipped with a Captive Spray nano electrospray source (Bruker Daltonics). Data were acquired using data-dependent auto-MS/MS with a 100-1700 m/z mass range, with parallel accumulation-serial fragmentation (PASEF) enabled and PASEF scans set at 10 (1.17 s duty cycle) and a dynamic exclusion of 0.4 min, m/z-dependent isolation window and collision energy of 42.0 eV. The intensity threshold was set at 2500, and the target intensity was set at 20,000.

### Protein identification for the DIA library (TIMS DDA)

Using the MaxQuant software (version 2.0.3.0; www.maxquant.org) and the UniProt mouse proteome database (February 2022, 55,286 entries), the raw files were analyzed to obtain the DIA library. The following settings were used for the MaxQuant analysis (with group-specific TIMS-DDA type parameters): one miscleavage was authorized; cysteine carbamidomethylation was a fixed modification; trypsin was the enzyme (lysine/arginine was not before a proline); methionine oxidation and protein N-terminal modification were included in the analysis; precursor and fragment ions had a mass tolerance of 20 ppm; ‘PSM FDR’, ‘protein FDR’ and ‘site decoy fraction’ identification values were set to 0.05; 1 was the minimum peptide count; ‘match between runs’ and ‘second peptides’ were permitted; and a transfer q-value of 0.3 was used to run MaxQuant. From this DDA analysis, the ‘peptides.txt’, ‘evidence.txt’ and ‘msms.txt’ files were used for DIA analysis.

### DIA LC-MS analysis

Data were acquired with the diaPASEF mode and using the parameters described for DDA analysis. One mobility window with 27 mass steps (m/z between 114 and 1414 with a mass width of 50 Da) per cycle (1.27 s duty cycle) was used for each single TIMS (100 ms) in diaPASEF mode, which covers in the m/z-ion mobility plane the diagonal scan line for charged peptides +2 and +3.

### Protein identification by DIA-MS

MaxQuant software (version 2.0.3.0) and UniProt mouse proteome database (02/2022, 55,286 entries) were used to analyze the raw files. The settings were as described above, except for the following: Maxquant and TIMS MaxDIA were selected; three files (‘peptides.txt’, ‘evidence.txt’ and ‘msms.txt’) from the DIA library were used.

### Data analysis for DIA-MS

Single peptides and proteins positive for the ‘reverse’, ‘only identified by site’ or ‘potential contaminant’ categories were excluded. Proteomic data were then analyzed using the iPathwayGuide software (Advaita Bio) with a threshold of 1.5 for log_2_ fold-change [calculated with flq (label-free quantification)-intensity] and 0.05 for *P*-value. GO terms for biological processes were corrected with a false discovery rate (FDR). Graphs were obtained using R software. Networks were obtained using the Cytoscape software. Proteomics data have been deposited to the ProteomeXchange with PRIDE repository partner (PXD039477).

### Bioinformatic analysis for putative binding sites

Alignment and putative binding sites were obtained using the Contra v3 web tool (Kreft et al., 2017). The following parameters were used: visualization, organism *Mus musculus*, gene *Ascl2* (NM_008554) and 500 bp promoter region. The stringency was as followed: core=0.95, similarity matrix=0.85. Alignment was performed on the ten most upregulated and downregulated proteins obtained by MS analysis.

### Statistical analysis

Statistical analysis and graphs were obtained using GraphPad Prism 9 software. The following statistical tests were performed: two-tailed paired and unpaired Student's *t*-test, two-tailed unpaired Welch's *t*-test and two-way. Tests were corrected for variance, multiple comparisons and normality. Error bars shown on graphs correspond to the standard deviation or standard error. The significance of the *P*-value is represented as follows: *P≤0.05, **P≤0.01, ***P≤0.001 and ****P≤0.0001.

## Supplementary Material

10.1242/biolopen.059919_sup1 Supplementary information

## References

[BIO059919C1] Asfaha, S., Hayakawa, Y., Muley, A., Stokes, S., Graham, T. A., Ericksen, R. E., Westphalen, C. B., von Burstin, J., Mastracci, T. L., Worthley, D. L. et al. (2015). Krt19(+)/Lgr5(−) cells are radioresistant cancer-initiating stem cells in the colon and intestine. *Cell Stem Cell* 16, 627-638. 10.1016/j.stem.2015.04.013 26046762 PMC4457942

[BIO059919C2] Ayyaz, A., Kumar, S., Sangiorgi, B., Ghoshal, B., Gosio, J., Ouladan, S., Fink, M., Barutcu, S., Trcka, D., Shen, J. et al. (2019). Single-cell transcriptomes of the regenerating intestine reveal a revival stem cell. *Nature* 569, 121-125. 10.1038/s41586-019-1154-y 31019301

[BIO059919C3] Bao, H., Liu, D., Xu, Y., Sun, Y., Mu, C., Yu, Y., Wang, C., Han, Q., Liu, S., Cai, H. et al. (2020). Hyperactivated Wnt-β-catenin signaling in the absence of sFRP1 and sFRP5 disrupts trophoblast differentiation through repression of Ascl2. *BMC Biol.* 18, 151. 10.1186/s12915-020-00883-4 33109217 PMC7592576

[BIO059919C4] Bogutz, A. B., Oh-Mcginnis, R., Jacob, K. J., Ho-Lau, R., Gu, T., Gertsenstein, M., Nagy, A. and Lefebvre, L. (2018). Transcription factor ASCL2 is required for development of the glycogen trophoblast cell lineage. *PLoS Genet.* 14, e1007587. 10.1371/journal.pgen.1007587 30096149 PMC6105033

[BIO059919C5] Busslinger, G. A., Weusten, B. L. A., Bogte, A., Begthel, H., Brosens, L. A. A. and Clevers, H. (2021). Human gastrointestinal epithelia of the esophagus, stomach, and duodenum resolved at single-cell resolution. *Cell Rep.* 34, 108819. 10.1016/j.celrep.2021.108819 33691112

[BIO059919C6] Croagh, D., Phillips, W. A., Redvers, R., Thomas, R. J. S. and Kaur, P. (2007). Identification of candidate murine esophageal stem cells using a combination of cell kinetic studies and cell surface markers. *Stem Cells* 25, 313-318. 10.1634/stemcells.2006-0421 17038667

[BIO059919C7] Croagh, D., Thomas, R. J. S., Phillips, W. A. and Kaur, P. (2008). Esophageal stem cells—a review of their identification and characterization. *Stem Cell Rev.* 4, 261-268. 10.1007/s12015-008-9031-3 18679835

[BIO059919C8] Deward, A. D., Cramer, J. and Lagasse, E. (2014). Cellular heterogeneity in the mouse esophagus implicates the presence of a nonquiescent epithelial stem cell population. *Cell Rep.* 9, 701-711. 10.1016/j.celrep.2014.09.027 25373907 PMC4223874

[BIO059919C9] Doupé, D. P., Alcolea, M. P., Roshan, A., Zhang, G., Klein, A. M., Simons, B. D. and Jones, P. H. (2012). A single progenitor population switches behavior to maintain and repair esophageal epithelium. *Science* 337, 1091-1093. 10.1126/science.1218835 22821983 PMC3527005

[BIO059919C10] Giakountis, A., Moulos, P., Zarkou, V., Oikonomou, C., Harokopos, V., Hatzigeorgiou, A. G., Reczko, M. and Hatzis, P. (2016). A positive regulatory loop between a Wnt-regulated non-coding RNA and ASCL2 controls intestinal stem cell fate. *Cell Rep.* 15, 2588-2596. 10.1016/j.celrep.2016.05.038 27292638

[BIO059919C11] Giroux, V., Lento, A. A., Islam, M., Pitaressi, J. R., Kharbanda, A., Hamilton, K. E., Whelan, K. A., Long, A., Rhoades, B., Tang, Q. et al. (2017). Long-lived keratin 15+ esophageal progenitor cells contribute to homeostasis and regeneration. *J. Clin. Invest.* 127, 2378-2391. 10.1172/JCI88941 28481227 PMC5451220

[BIO059919C12] Giroux, V., Stephan, J., Chatterji, P., Rhoades, B., Wileyto, E. P., Klein-Szanto, A. J., Lengner, C. J., Hamilton, K. E. and Rustgi, A. K. (2018). Mouse intestinal Krt15+ crypt cells are radio-resistant and tumor initiating. *Stem Cell Reports* 10, 1947-1958. 10.1016/j.stemcr.2018.04.022 29805107 PMC5993649

[BIO059919C13] Hua, G., Thin, T. H., Feldman, R., Haimovitz–Friedman, A., Clevers, H., Fuks, Z. and Kolesnick, R. (2012). Crypt base columnar stem cells in small intestines of mice are radioresistant. *Gastroenterology* 143, 1266-1276. 10.1053/j.gastro.2012.07.106 22841781 PMC3480544

[BIO059919C14] Jiang, M., Ku, W.-Y., Zhou, Z., Dellon, E. S., Falk, G. W., Nakagawa, H., Wang, M.-L., Liu, K., Wang, J., Katzka, D. A. et al. (2015). BMP-driven NRF2 activation in esophageal basal cell differentiation and eosinophilic esophagitis. *J. Clin. Invest.* 125, 1557-1568. 10.1172/JCI78850 25774506 PMC4396468

[BIO059919C15] Kalabis, J., Oyama, K., Okawa, T., Nakagawa, H., Michaylira, C. Z., Stairs, D. B., Figueiredo, J.-L., Mahmood, U., Diehl, J. A., Herlyn, M. et al. (2008). A subpopulation of mouse esophageal basal cells has properties of stem cells with the capacity for self-renewal and lineage specification. *J. Clin. Invest.* 118, 3860-3869. 10.1172/JCI35012 19033657 PMC2579884

[BIO059919C16] Kinoshita, M., Li, M. A., Barber, M., Mansfield, W., Dietmann, S. and Smith, A. (2021). Disabling de novo DNA methylation in embryonic stem cells allows an illegitimate fate trajectory. *Proc. Natl. Acad. Sci. USA* 118, e2109475118. 10.1073/pnas.2109475118 34518230 PMC8463881

[BIO059919C17] Kreft, Ł., Kreft, Ł., Soete, A., Hulpiau, P., Botzki, A., Saeys, Y. and De Bleser, P. (2017). ConTra v3: a tool to identify transcription factor binding sites across species, update 2017. *Nucleic Acids Res.* 45, W490-W494. 10.1093/nar/gkx376 28472390 PMC5570180

[BIO059919C18] Küry, P., Greiner-Petter, R., Cornely, C., Jürgens, T. and Müller, H. W. (2002). Mammalian achaete scute homolog 2 is expressed in the adult sciatic nerve and regulates the expression of Krox24, Mob-1, CXCR4, and p57kip2 in Schwann cells. *J. Neurosci.* 22, 7586-7595. 10.1523/JNEUROSCI.22-17-07586.2002 12196582 PMC6758000

[BIO059919C19] Kwon, O.-H., Park, J.-L., Baek, S.-J., Noh, S.-M., Song, K.-S., Kim, S.-Y. and Kim, Y. S. (2013). Aberrant upregulation of ASCL2 by promoter demethylation promotes the growth and resistance to 5-fluorouracil of gastric cancer cells. *Cancer Sci.* 104, 391-397. 10.1111/cas.12076 23181270 PMC7657231

[BIO059919C20] Liu, X., Chen, X., Zhong, B., Wang, A., Wang, X., Chu, F., Nurieva, R. I., Yan, X., Chen, P., van der Flier, L. G. et al. (2014). Transcription factor achaete-scute homologue 2 initiates follicular T-helper-cell development. *Nature* 507, 513-518. 10.1038/nature12910 24463518 PMC4012617

[BIO059919C21] Liu, Z., Wang, X., Jiang, K., Ji, X., Zhang, Y. A. and Chen, Z. (2019). TNFα-induced up-regulation of Ascl2 affects the differentiation and proliferation of neural stem cells. *Aging Dis.* 10, 1207-1220. 10.14336/AD.2018.1028 31788333 PMC6844591

[BIO059919C22] Luo, A., Chen, H., Ding, F., Zhang, Y., Wang, M., Xiao, Z. and Liu, Z. (2013). Small proline-rich repeat protein 3 enhances the sensitivity of esophageal cancer cells in response to DNA damage-induced apoptosis. *Mol. Oncol.* 7, 955-967. 10.1016/j.molonc.2013.05.005 23820115 PMC5528455

[BIO059919C23] Medema, R. H., Herrera, R. E., Lam, F. and Weinberg, R. A. (1995). Growth suppression by p16ink4 requires functional retinoblastoma protein. *Proc. Natl. Acad. Sci. USA* 92, 6289-6293. 10.1073/pnas.92.14.6289 7603984 PMC41503

[BIO059919C24] Moriyama, M., Durham, A.-D., Moriyama, H., Hasegawa, K., Nishikawa, S.-I., Radtke, F. and Osawa, M. (2008). Multiple roles of Notch signaling in the regulation of epidermal development. *Dev. Cell* 14, 594-604. 10.1016/j.devcel.2008.01.017 18410734

[BIO059919C25] Murata, K., Jadhav, U., Madha, S., Van Es, J., Dean, J., Cavazza, A., Wucherpfennig, K., Michor, F., Clevers, H. and Shivdasani, R. A. (2020). Ascl2-dependent cell dedifferentiation drives regeneration of ablated intestinal stem cells. *Cell Stem Cell* 26, 377-390.e6. 10.1016/j.stem.2019.12.011 32084390 PMC7147146

[BIO059919C26] Ohashi, S., Natsuizaka, M., Yashiro–Ohtani, Y., Kalman, R. A., Nakagawa, M., Wu, L., Klein–Szanto, A. J., Herlyn, M., Diehl, J. A., Katz, J. P. et al. (2010). NOTCH1 and NOTCH3 coordinate esophageal squamous differentiation through a CSL-dependent transcriptional network. *Gastroenterology* 139, 2113-2123. 10.1053/j.gastro.2010.08.040 20801121 PMC2997138

[BIO059919C27] Oomizu, S., Sahuc, F., Asahina, K., Inamatsu, M., Matsuzaki, T., Sasaki, M., Obara, M. and Yoshizato, K. (2000). Kdap, a novel gene associated with the stratification of the epithelium. *Gene* 256, 19-27. 10.1016/S0378-1119(00)00357-7 11054531

[BIO059919C28] Rosekrans, S. L., Baan, B., Muncan, V. and Van Den Brink, G. R. (2015). Esophageal development and epithelial homeostasis. *Am. J. Physiol. Gastrointest. Liver Physiol.* 309, G216-G228. 10.1152/ajpgi.00088.2015 26138464

[BIO059919C29] Schuijers, J., Junker, J. P., Mokry, M., Hatzis, P., Koo, B.-K., Sasselli, V., van der Flier, L. G., Cuppen, E., van Oudenaarden, A. and Clevers, H. (2015). Ascl2 acts as an R-spondin/Wnt-responsive switch to control stemness in intestinal crypts. *Cell Stem Cell* 16, 158-170. 10.1016/j.stem.2014.12.006 25620640

[BIO059919C30] Shibahara, Y., Espin-Garcia, O., Conner, J., Weiss, J., Derouet, M., Allen, J., Allison, F., Kalimuthu, S., Yeung, J. C. and Darling, G. E. (2022). Intestinal stem cell marker ASCL2 is a novel prognostic predictor in esophageal adenocarcinoma. *Cureus* 14, e21021. 10.7759/cureus.21021 35154991 PMC8818334

[BIO059919C31] Squier, C. A. and Kremer, M. J. (2001). Biology of oral mucosa and esophagus. *J. Natl. Cancer Inst. Monogr.* 2001, 7-15. 10.1093/oxfordjournals.jncimonographs.a003443 11694559

[BIO059919C32] Tanaka, T., Kojima, K., Yokota, K., Tanaka, Y., Ooizumi, Y., Ishii, S., Nishizawa, N., Yokoi, K., Ushiku, H., Kikuchi, M. et al. (2019). Comprehensive genetic search to clarify the molecular mechanism of drug resistance identifies ASCL2-LEF1/TSPAN8 axis in colorectal cancer. *Ann. Surg. Oncol.* 26, 1401-1411. 10.1245/s10434-019-07172-7 30706227

[BIO059919C33] van der Flier, L. G., Van Gijn, M. E., Hatzis, P., Kujala, P., Haegebarth, A., Stange, D. E., Begthel, H., Van Den Born, M., Guryev, V., Oving, I. et al. (2009). Transcription factor achaete scute-like 2 controls intestinal stem cell fate. *Cell* 136, 903-912. 10.1016/j.cell.2009.01.031 19269367

[BIO059919C34] Varberg, K. M., Iqbal, K., Muto, M., Simon, M. E., Scott, R. L., Kozai, K., Choudhury, R. H., Aplin, J. D., Biswell, R., Gibson, M. et al. (2021). ASCL2 reciprocally controls key trophoblast lineage decisions during hemochorial placenta development. *Proc. Natl. Acad. Sci. USA* 118, e2016517118. 10.1073/pnas.2016517118 33649217 PMC7958375

[BIO059919C35] Wang, C., Wang, M., Arrington, J., Shan, T., Yue, F., Nie, Y., Tao, W. A. and Kuang, S. (2017). Ascl2 inhibits myogenesis by antagonizing the transcriptional activity of myogenic regulatory factors. *Development* 144, 235-247. 10.1242/dev.138099 27993983 PMC5394758

[BIO059919C36] Zakrzewski, W., Dobrzyński, M., Szymonowicz, M. and Rybak, Z. (2019). Stem cells: past, present, and future. *Stem Cell Res. Ther.* 10, 68. 10.1186/s13287-019-1165-5S 30808416 PMC6390367

