# Supplementary material for: ASCL2 is a key regulator of the proliferation–differentiation equilibrium in the esophageal epithelium
Source: Biol Open. 2024 Jan 22;13(1):bio059919. doi: 10.1242/bio.059919 (PMC10836648; doi:10.1242/bio.059919)
Supplement: Supplementary information [file biolopen-13-059919-s1.pdf]

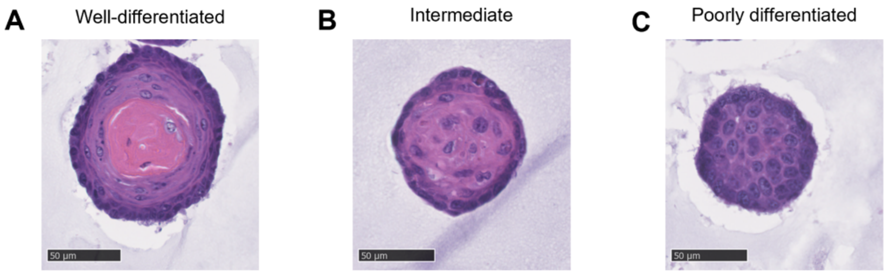

Fig. S1. Representative image of H&E coloration of organoids in each category of differentiation.

(A) Well-differentiated organoids reproduce completely the normal esophageal epithelium with a keratinized center. **(B)** Organoids in the intermediate category possess basal cells (well aligned and nearly only the nucleus is visible) and suprabasal cells (more flat than basal cell, with cytoplasm more visible) but are missing the keratinized center. **(C)** Poorly differentiated organoids are composed only of basal cells in appearance.

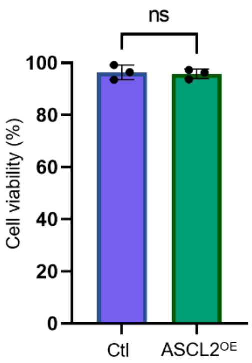

Fig. S2. **Cell viability is not modified by overexpression of ASCL2.**

Cell viability was assessed using Vivafix^{TM} in flow cytometry. Graph represents mean ± SD (n = 3).

Biology Open • Supplementary information

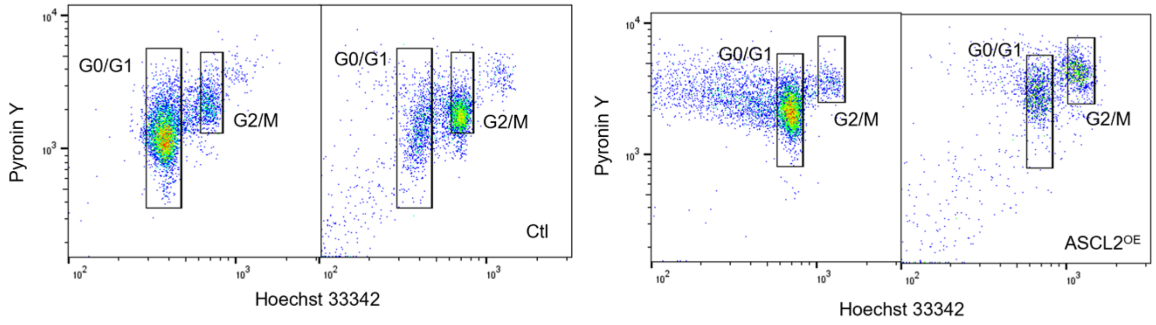

Fig. S3. Nocodazole treatment to confirm cell cycle phase.

Ctl and ASCL2^{OE} organoids were treated with 100 ng/mL nocodazole (right panel) or without (left panel, n = 3) for 24 hours. Cell cycle was analysed by flow cytometry as in Fig. 3. Representative data is shown.

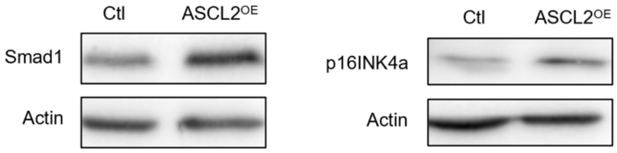

Fig. S4. Validation of proteomic analysis.

Smad1 and p16INK4a increase in proteomic data was confirmed in ASCL2OE organoids by Western Blot (n=4). Representative sample is shown.

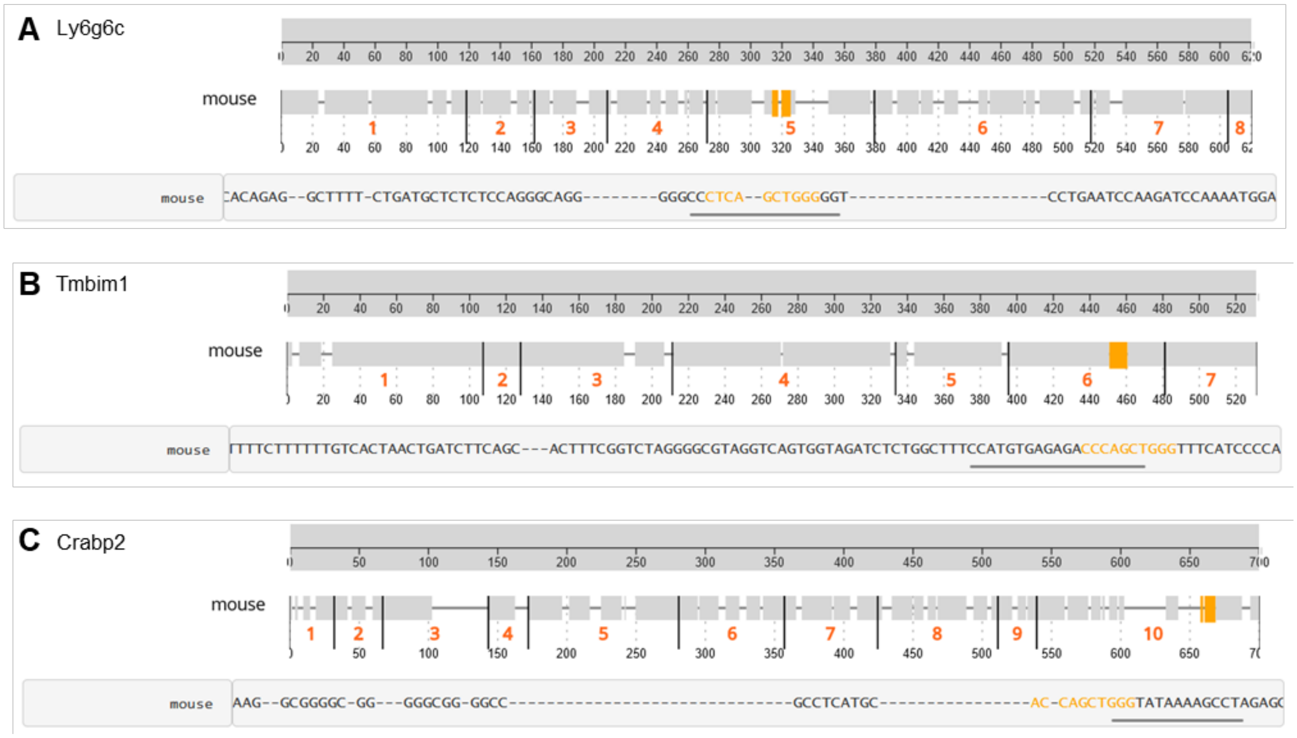

Fig. S5. Putative binding sites for ASCL2 on the promoter of MS obtained genes.

Exploration of putative binding sites for ASCL2 (MA0816.1 from JASPAR CORE 2016 database) in the 500bp promoter region of genes encoding for proteins that were downregulated in ASCL2^{OE} organoids. The alignment was obtained using ConTra v3 with the following stringency: core = 0,95, similarity matrix = 0,85. Binding sites are highlighted in yellow-orange. **(A)** Alignment for the promoter region of the gene *Ly6g6c*. **(B)** Alignment for the promoter region of the gene *Tmbim1*. **(C)** Alignment for the promoter region of the gene *Crabp2*.

Biology Open • Supplementary information

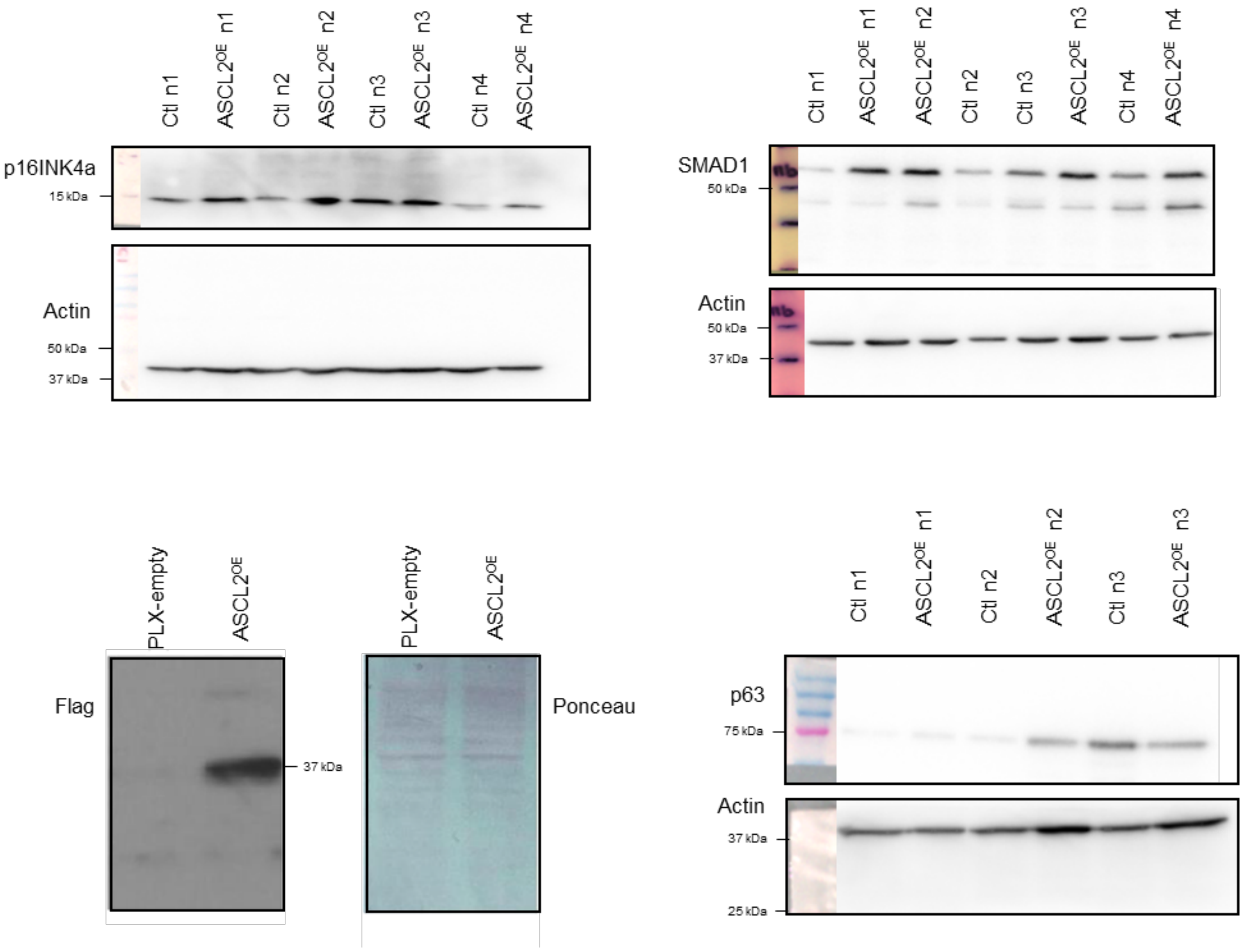

Fig. S6. Complete membranes for the WB
